# Synthesis, crystal structure and Hirshfeld analysis of a novel supra­molecular compound [Co(tsc)_3_]_2_[Co(cit)_2_](NO_3_)_4_·4H_2_O

**DOI:** 10.1107/S2056989025001136

**Published:** 2025-02-18

**Authors:** Guzal Nuralieva, Oydinoy Umirzakova, Batirbay Torambetov, Abdusamat Rasulov, Jamshid Ashurov, Shakhnoza Kadirova

**Affiliations:** a National University of Uzbekistan named after Mirzo Ulugbek 4 University St Tashkent 100174 Uzbekistan; b Physical and Materials Chemistry Division CSIR-National Chemical Laboratory,Pune-411008 India; c Termez University of Economics and Service, 41B Farovon St, Termiz, 190111, Uzbekistan; d Institute of Bioorganic Chemistry, Academy of Sciences of Uzbekistan, M. Ulugbek St, 83, Tashkent, 100125, Uzbekistan; e National University of Uzbekistan named after Mirzo Ulugbek 4 University St Tashkent 100174 Uzbekistan; Universidade de Sâo Paulo, Brazil

**Keywords:** crystal structure, cobalt, thio­semicarbazide, citric acid, hydrogen bonding, Hirshfeld analysis

## Abstract

A new cobalt complex, bis­[tris­(amino­thio­urea)cobalt(III)] bis­[2-(carb­oxy­methyl)-2-hy­droxy­butane­dioato]cobalt(II) tetra­nitrate tetra­hydrate, [Co(CH_5_N_3_S)_3_][Co(C_6_H_6_O_7_)_2_]_0.5_(NO_3_)_2_·2H_2_O, designated as [Co(tsc)_3_]_2_[Co(cit)_2_](NO_3_)_4_·4H_2_O, was synthesized. Two crystallographically independent cobalt centers are present. In the first, the central metal atom is chelated by three thio­semicarbazide ligands in a bidentate fashion whereas the second, positioned on a crystallographic inversion center, is hexa­coordinated by two citrate anions in a distorted octa­hedral geometry. Additionally, two water mol­ecules and two nitrate anions are present in the asymmetric unit. Hirshfeld surface analysis revealed that the presence of numerous donor and acceptor groups in the complex, which facilitate hydrogen-bonding inter­actions that contribute significantly to the overall cohesion of the crystal structure.

## Abstract 2

The mol­ecular and crystal structures of the coordination compound [Co(tsc)_3_]_2_[Co(cit)_2_](NO_3_)_4_·4H_2_O are reported. Fingerprint plots were generated to investigate various inter­molecular inter­actions.

## 1. Chemical context

Thio­semicarbazide is a widely used ligand in coordination chemistry because of its strong complex-forming ability, attributed to the presence of sulfur and nitro­gen donor atoms, which enables it to act as a bidentate ligand (Ibrahim & Bekheit, 1988 ▸). Its coordination with transition metals is particularly inter­esting, as these metals can adopt diverse geometries such as tetra­hedral, square-planar, and octa­hedral depending on the surrounding ligands, enhancing the stability and versatility of the resulting complexes (Hussain, 1994 ▸; Yang *et al.*, 2006 ▸; Burrows *et al.*, 1997 ▸). Citric acid, a tri­carb­oxy­lic acid, is another versatile mol­ecule known for its role in both chemistry and biology. The citrate dianion acts as a multi-dentate ligand, coordinating with metals through carboxyl­ate (–COO^−^) and hydroxyl (–OH) groups, allowing for the formation of robust metal complexes. The applications of complexes formed from thio­semicarbazide and citric acid can be found in catalysis, biomedicine, and environmental remediation (Koolivand *et al.*, 2021 ▸; Wakizaka *et al.*, 2024 ▸; Andres *et al.*, 2020 ▸; Singh *et al.*, 2023 ▸).

Although extensive research has been conducted on thio­semicarbazide complexes with metals such as nickel, cobalt, and zinc, systems containing two crystallographically independent centers remain underexplored (Konarev *et al.*, 2004 ▸; Antsyshkina *et al.*, 2014 ▸). In this work, cobalt was selected as the central metal ion, and citric acid was utilized as a multifunctional ligand to construct a supra­molecular framework. This combination provides an excellent model to study the inter­play of metal–ligand coordination and hydrogen-bonding networks. We report the synthesis and crystal structure of a new cobalt complex, [Co(tsc)_3_]_2_[Co(cit)_2_](NO_3_)_4_·4H_2_O, and highlight the role of citric acid as a key component in the assembly of hybrid materials.
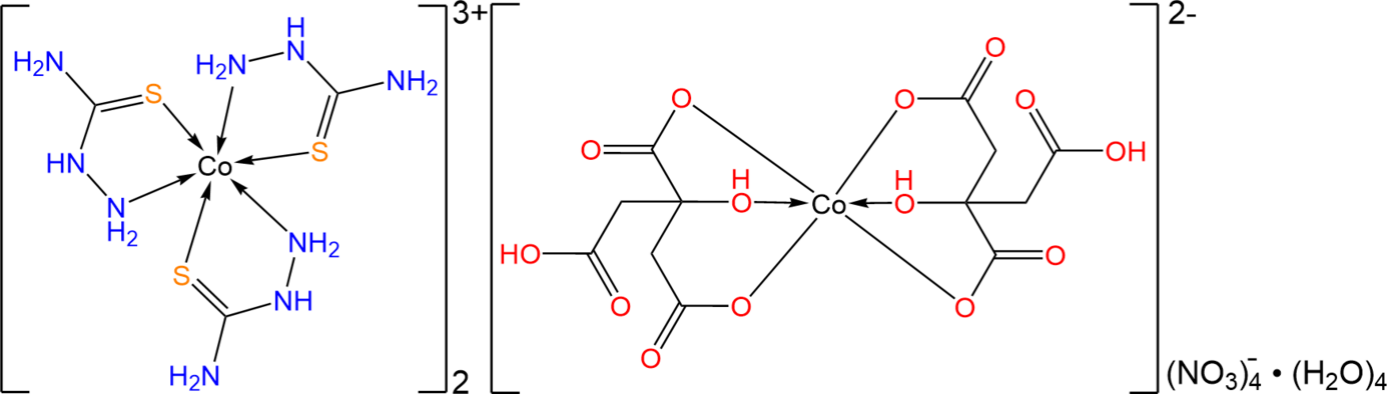


## 2. Structural commentary

**Figure 1:**
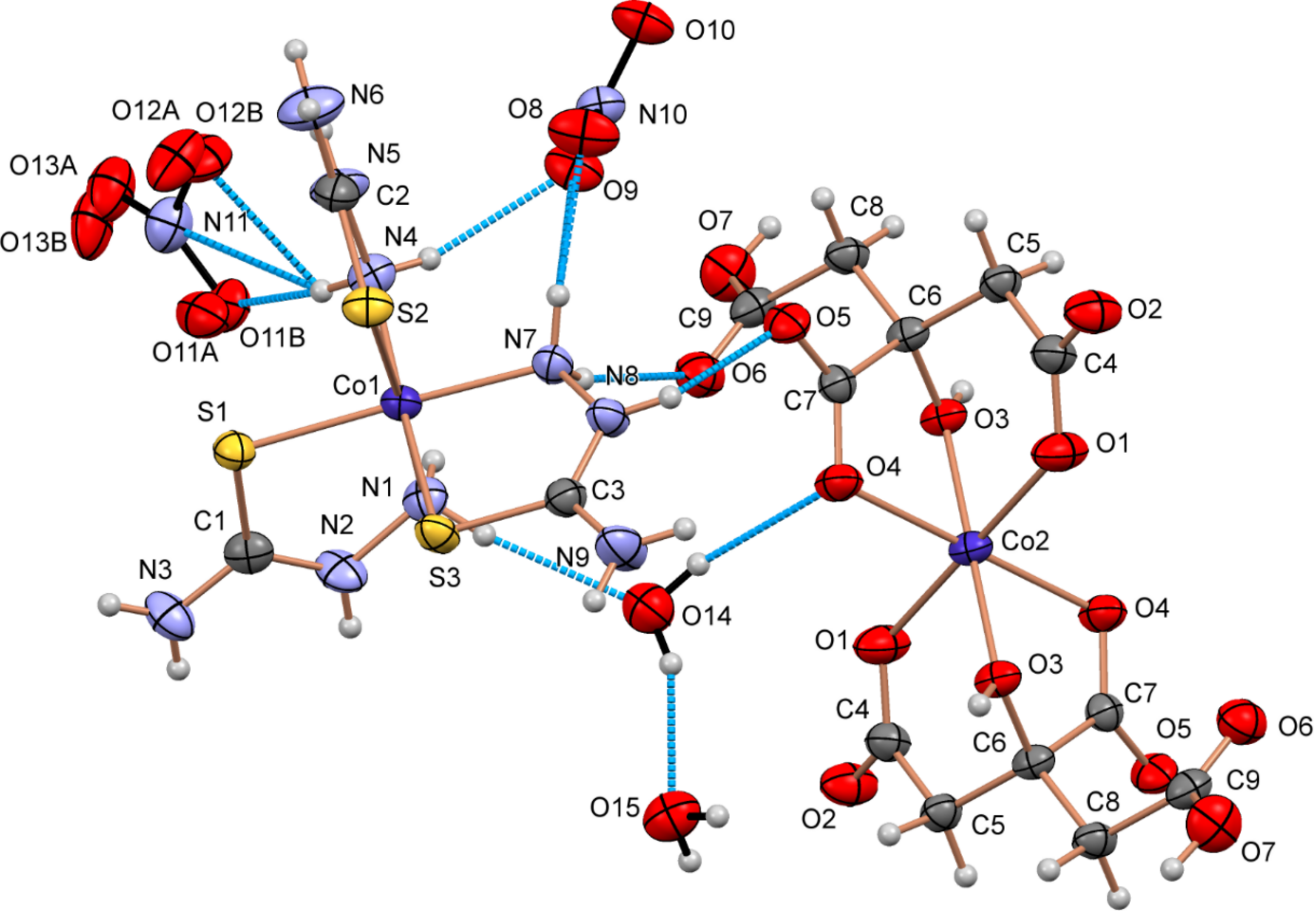
[Co(tsc)_3_]_2_[Co(cit)_2_](NO_3_)_4_·4H_2_O with displacement ellipsoids drawn at the 50% probability level and hydrogen atoms shown as small spheres. Intra­mol­ecular hydrogen bonds are indicated by dotted lines.

The structure of [Co(tsc)_3_]_2_[Co(cit)_2_](NO_3_)_4_·4H_2_O is shown in Fig. 1 ▸. The complex crystallizes in the monoclinic system with a *P*2_1_/*c* space group with two crystallographically independent cobalt centers, namely, [Co(tsc)_3_] and [Co(cit)_2_], designated as CoC1 and CoC2, respectively. The asymmetric unit of [Co(tsc)_3_]_2_[Co(cit)_2_](NO_3_)_4_·4H_2_O comprises one mol­ecule of CoC1, a half-mol­ecule of CoC2, two water mol­ecules, and two nitrate anions (CoC1 and CoC2 are in a 2:1 ratio). The cobalt centers exhibit different oxidation states, with the cobalt atom in CoC1 being in the +3 oxidation state, while in CoC2, it is in the +2 oxidation state. In the first cobalt center (CoC1), the cobalt(III) atom is coordinated by three thio­semicarbazide ligands in a bidentate manner, involving nitro­gen and sulfur donor atoms and resulting in the formation of three five-membered rings. The Co—N bond lengths are in the range 1.991 (3)–2.002 (4) Å, while the Co—S bond length are 2.1967 (11)–2.2265 (11) Å. The cobalt(II) atom in the second cobalt center (CoC2) is tridentately chelated by the two citrate ligand through three oxygen atoms from each ligand, *i.e*. two from carboxyl­ate groups and one from a hydroxyl group of the citrate dianion, forming two five-membered and two six-membered rings around the central metal atom. The central cobalt atom exhibits a distorted octa­hedral geometry and occupies special position on the inversion center. The Co—O (carboxyl­ate) bond lengths are in the range 2.081 (3)–2.084 (4) Å, and the Co—O (hydrox­yl) bond length is 2.060 (3) Å. Two mol­ecules of water and a nitrate anion remain uncoordinated in the asymmetric unit but are involved in inter­actions with both the cobalt centers and assist in the formation of supra­mol­ecular construct.

## 3. Supra­molecular features

In the title complex, the complex cations and the water mol­ecule have proton-donor hydrogen-bonding groups, whereas the oxygen atoms of the nitrate anion and citrate ligands act as proton acceptors in an intricate network of hydrogen bonds (Table 1 ▸). The nitrate anions participate in strong hydrogen bonding with the amine hydrogen atoms (N4—H4*B*⋯O12*A* and N7—H7*A*⋯O6) of the coordinated thio­semicarbazide ligands. Furthermore, hydrogen bonding is observed between the two cobalt centers. This involves inter­actions between the amine hydrogens (N7—H7*A*⋯O6 and N8—H8⋯O5) of the thio­semicarbazide ligands from CoC1 and the oxygen atoms of the citrate ligand in the adjacent CoC2 center.

**Figure 2:**
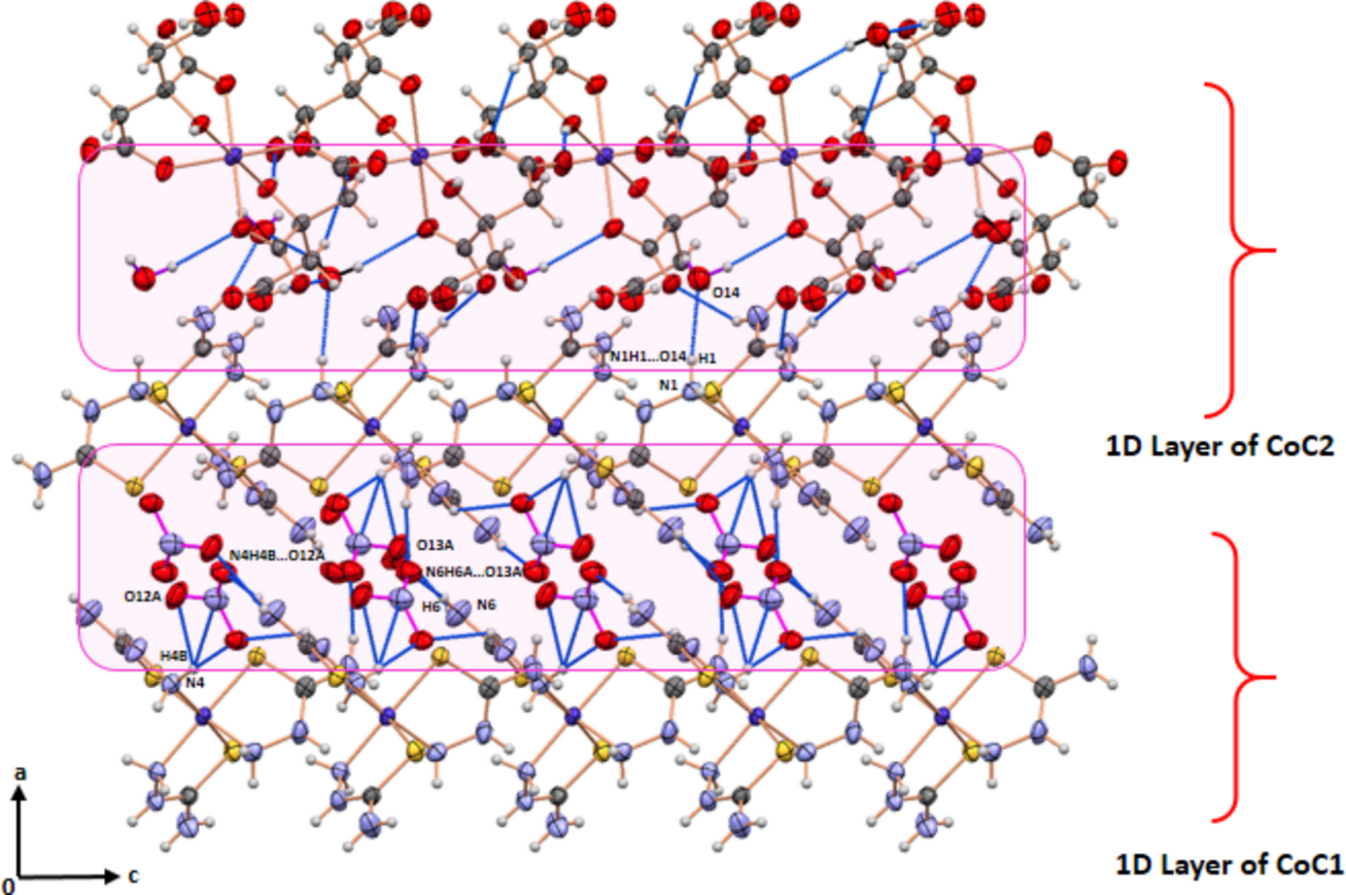
View of the packing of mol­ecules in [Co(tsc)_3_]_2_[Co(cit)_2_](NO_3_)_4_·4H_2_O along the *b* axis.

The two uncoordinated water mol­ecules form hydrogen bonds with each other, while also inter­acting with the amine nitro­gen (N1—H1*B*⋯O14) of the thio­semicarbazide ligand and a neighboring oxygen atom (O14—H14*A*⋯O4) of the citrate dianion in the second cobalt center (Fig. 1 ▸). The nitrate anions are located between two CoC1 layers, whereas the water mol­ecules placed between the CoC1 and CoC2 layers. Thus, both the nitrate anions and the water mol­ecules contribute significantly to the structure of the complex by forming an extensive hydrogen-bond network and form a 1D layered assembly parallel to the *c*-axis direction. Although the hydrogen bonds are relatively weak, all potential donors and acceptors participate, providing notable cohesion to the overall structure (Fig. 2 ▸).

## 4. Hirshfeld Surface Analysis

**Figure 3:**
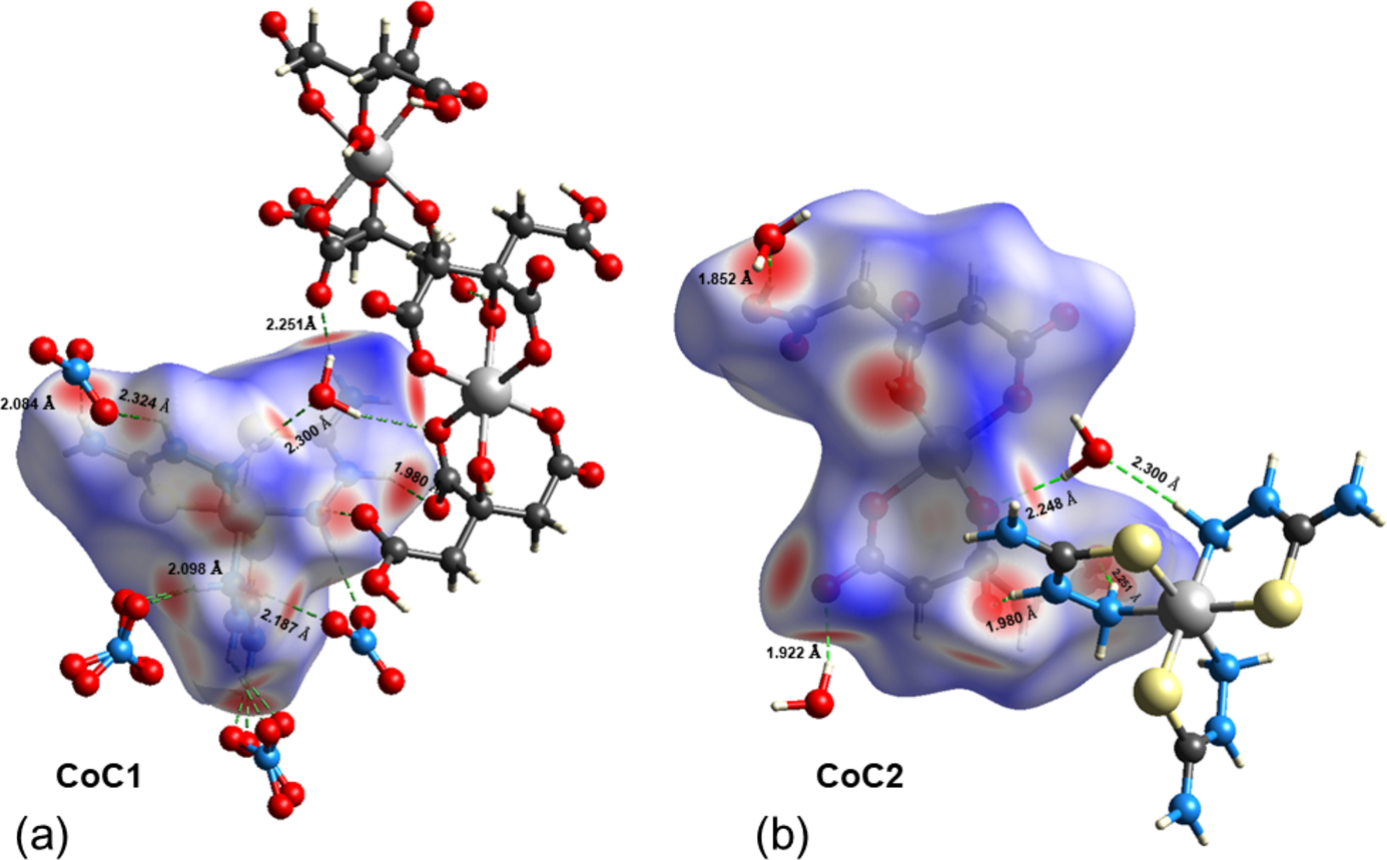
The three-dimensional Hirshfeld surfaces for (*a*) CoC1 and (*b*) CoC2 in [Co(tsc)_3_]_2_[Co(cit)_2_](NO_3_)_4_·4H_2_O.

Hirshfeld surface (Spackman & Jayatilaka, 2009 ▸) analysis and fingerprint plot analysis (Spackman & McKinnon, 2002 ▸) were performed using *CrystalExplorer21.5* (Wolff *et al.*, 2012 ▸) to investigate the inter­molecular inter­actions. These were both performed separately for CoC1 and CoC2, as shown in Figs. 3 ▸ and 4 ▸. The red spots on the Hirshfeld surface are due to short O⋯H inter­actions, which are mapped on the 2D fingerprint plots. The mol­ecule exhibits a significant number of hydrogen-bonding inter­actions, with O⋯H/H⋯O, H⋯H, S⋯H/H⋯S, S⋯O/O⋯S, and N⋯H/H⋯N inter­actions accounting for 85.7% of the total inter­actions in CoC1. In contrast, inter­actions such as C⋯S, N⋯S, and S⋯S, play a minor role in the crystal cohesion. However, O⋯H/H⋯O, H⋯H, O⋯O, C⋯H/H⋯C and N⋯H/H⋯N contacts represent 99.2% of the total inter­actions in CoC2 (Figs. 3 ▸, 4 ▸).

## 5. Database survey

A survey of the Cambridge Structural Database (CSD, Version 5.45, last updated March 2024; Groom *et al.*, 2016 ▸) revealed that seven crystal structures for cobalt complexes with three thio­semicarbazide ligands have been reported [AZILOL (Liu *et al.*, 2009 ▸); BAYGUD (Rusanovskii *et al.*, 1981 ▸); GEWNIF (Larsen *et al.*, 1988 ▸); KAZBAP (Bulimestru *et al.*, 2005 ▸); THSMCB (Samus *et al.*, 1981 ▸); WEWFUZ (Hussain, 1994 ▸); YUNTUW (Zhang *et al.*, 1994 ▸)]. The CSD includes around 40 structures where citric acid is directly bonded to a cobalt atom, in only three of which [ADENAY, (Herynek *et al.*, 2000 ▸); IDANOR (Galloway *et al.*, 2006 ▸); QEQVAJ (Shvelashvili *et al.*, 2000 ▸)] are two citric acid ligands bonded tridentately to form a hexa­coordinated complex. However, no complexes containing both citric acid and thio­semicarbazide with two different coordination centers have been reported.

## 6. Synthesis and crystallization

Co(NO_3_)_2_·6H_2_O (0. 291 g, 1 mmol), thio­semicarbazide (0.091 g, 1 mmol) and citric acid (0.192 g, 1 mmol) were dissolved separately in 70% ethanol (5 ml), mixed together and stirred for 2 h at 333 K. The obtained pink solution was filtered and left for crystallization. Single crystals of the title complex suitable for X-ray analysis were obtained by slow evaporation of the solution over a period of 10 days (yield: 60%).

## 7. Refinement

Crystal data, data collection and structure refinement details are summarized in Table 2 ▸. All the hydrogen atoms were located in difference-Fourier maps and reﬁned using an isotropic approximation.

## Supplementary Material

Crystal structure: contains datablock(s) I. DOI: 10.1107/S2056989025001136/ex2089sup1.cif

Structure factors: contains datablock(s) I. DOI: 10.1107/S2056989025001136/ex2089Isup2.hkl

CCDC reference: 2364098

Additional supporting information:  crystallographic information; 3D view; checkCIF report

## Figures and Tables

**Figure 4:**
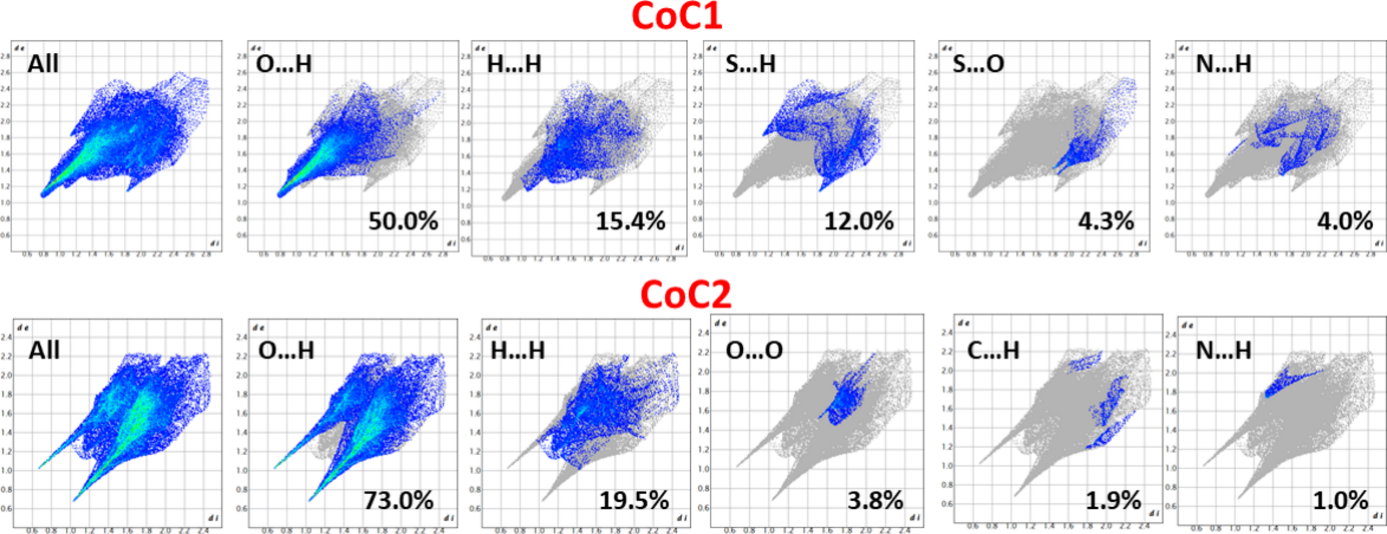
Two-dimensional fingerprint plots of the Hirshfeld surfaces showing contributions of various contacts for CoC1 (upper row) and CoC2 (lower row) in [Co(tsc)_3_]_2_[Co(cit)_2_](NO_3_)_4_·4H_2_O.

**Table 1:** Hydrogen-bond geometry (Å, °)

*D*—H⋯*A*	*D*—H	H⋯*A*	*D*⋯*A*	*D*—H⋯*A*
O14—H14*B*⋯O15	0.85	2.00	2.798 (5)	157
N7—H7*A*⋯O6	0.89	2.25	3.100 (5)	159
N7—H7*B*⋯O8	0.89	2.10	2.983 (5)	172
O7—H7⋯O14^i^	0.82	1.85	2.648 (5)	163
O15—H15*A*⋯O2^ii^	0.85	1.92	2.730 (5)	158
O15—H15*B*⋯O4^iii^	0.85	2.50	2.902 (5)	110
N1—H1*A*⋯O9^iv^	0.89	2.20	2.912 (5)	137
N1—H1*A*⋯O10^iv^	0.89	2.26	3.128 (5)	165
N1—H1*B*⋯O14	0.89	2.30	3.185 (5)	172
N8—H8⋯O5	0.86	1.98	2.670 (5)	136
N4—H4*A*⋯O9	0.89	2.19	3.000 (5)	152
N4—H4*A*⋯O10^iv^	0.89	2.45	3.024 (5)	122
N4—H4*B*⋯O11*A*	0.89	2.09	2.96 (3)	166
N4—H4*B*⋯O12*A*	0.89	2.48	3.06 (3)	123
N4—H4*B*⋯O12*B*	0.89	2.56	3.13 (2)	122
N4—H4*B*⋯O11*B*	0.89	2.15	3.04 (3)	175
N2—H2⋯O10^ii^	0.86	2.32	3.032 (5)	140
N5—H5⋯O11*A*^i^	0.86	2.28	2.96 (3)	136
N5—H5⋯O12*A*	0.86	2.47	2.92 (3)	114
N5—H5⋯O12*B*	0.86	2.45	2.88 (2)	112
N5—H5⋯O11*B*^i^	0.86	2.27	3.02 (3)	145
N3—H3*A*⋯O8^ii^	0.86	2.08	2.913 (5)	161
N3—H3*B*⋯O13*A*^v^	0.86	2.01	2.86 (3)	170
N3—H3*B*⋯O13*B*^v^	0.86	2.13	2.94 (3)	157
N9—H9*A*⋯O15^vi^	0.86	2.06	2.883 (6)	161
N9—H9*B*⋯O5^iii^	0.86	2.15	2.844 (5)	137
N6—H6*A*⋯O13*A*^i^	0.86	2.00	2.85 (3)	170
N6—H6*A*⋯O13*B*^i^	0.86	2.03	2.86 (3)	161
N6—H6*B*⋯O12*A*^vii^	0.86	2.35	2.93 (3)	125
N6—H6*B*⋯O12*B*^vii^	0.86	2.32	2.99 (3)	134

Symmetry codes: (i) 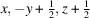; (ii) 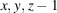; (iii) 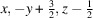; (iv) 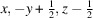; (v) 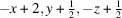; (vi) 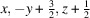; (vii) 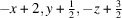.

**Table 2:** Experimental details

Crystal data	
Chemical formula	[Co(CH_5_N_3_S)_3_][Co(C_6_H_6_O_7_)_2_]_0.5_(NO_3_)_2_·2H_2_O
*M*_r_	711.97
Crystal system, space group	Monoclinic, *P*2_1_/*c*
Temperature (K)	293
*a*, *b*, *c* (Å)	22.8868 (7), 10.7978 (3), 10.1946 (3)
β (°)	95.359 (3)
*V* (Å^3^)	2508.35 (13)
*Z*	4
Radiation type	Cu *K*α
μ (mm^−1^)	11.05
Crystal size (mm)	0.12 × 0.08 × 0.06

Data collection	
Diffractometer	XtaLAB Synergy, Single source at home/near, HyPix3000
Absorption correction	Multi-scan (*CrysAlis PRO*; Rigaku OD, 2021 ▸)
*T*_min_, *T*_max_	0.419, 1.000
No. of measured, independent and observed [*I* > 2σ(*I*)] reflections	23912, 4582, 3715
*R*_int_	0.113
(sin θ/λ)_max_ (Å^−1^)	0.602

Refinement	
*R*[*F*^2^ > 2σ(*F*^2^)], *wR*(*F*^2^), *S*	0.055, 0.150, 1.00
No. of reflections	4582
No. of parameters	395
No. of restraints	3
H-atom treatment	H atoms treated by a mixture of independent and constrained refinement
Δρ_max_, Δρ_min_ (e Å^−3^)	0.92, −0.80

Computer programs: *CrysAlis PRO* (Rigaku OD, 2021 ▸), *SHELXT2018/2* (Sheldrick, 2015*a* ▸), *SHELXL2016/6* (Sheldrick, 2015*b* ▸) and *OLEX2* (Dolomanov *et al.*, 2009 ▸).

## supplementary crystallographic information

### Bis[tris(aminothiourea)cobalt(III)] bis[2-(carboxymethyl)-2-hydroxybutanedioato]cobalt(II) tetranitrate tetrahydrate Crystal data

**Table:**

[Co(CH_5_N_3_S)_3_][Co(C_6_H_6_O_7_)_2_]_0.5_(NO_3_)_2_·2H_2_O	*F*(000) = 1458
*M**_r_* = 711.97	*D*_x_ = 1.885 Mg m^−^^3^
Monoclinic, *P*2_1_/*c*	Cu *K*α radiation, λ = 1.54184 Å
*a* = 22.8868 (7) Å	Cell parameters from 6201 reflections
*b* = 10.7978 (3) Å	θ = 3.9–71.3°
*c* = 10.1946 (3) Å	µ = 11.05 mm^−^^1^
β = 95.359 (3)°	*T* = 293 K
*V* = 2508.35 (13) Å^3^	Block, red
*Z* = 4	0.12 × 0.08 × 0.06 mm

### Bis[tris(aminothiourea)cobalt(III)] bis[2-(carboxymethyl)-2-hydroxybutanedioato]cobalt(II) tetranitrate tetrahydrate Data collection

**Table:**

XtaLAB Synergy, Single source at home/near, HyPix3000 diffractometer	4582 independent reflections
Radiation source: micro-focus sealed X-ray tube, PhotonJet (Cu) X-ray Source	3715 reflections with *I* > 2σ(*I*)
Mirror monochromator	*R*_int_ = 0.113
Detector resolution: 10.0000 pixels mm^-1^	θ_max_ = 68.2°, θ_min_ = 3.9°
ω scans	*h* = −27→27
Absorption correction: multi-scan (CrysAlisPro; Rigaku OD, 2021)	*k* = −13→12
*T*_min_ = 0.419, *T*_max_ = 1.000	*l* = −12→9
23912 measured reflections	

### Bis[tris(aminothiourea)cobalt(III)] bis[2-(carboxymethyl)-2-hydroxybutanedioato]cobalt(II) tetranitrate tetrahydrate Refinement

**Table:**

Refinement on *F*^2^	Primary atom site location: dual
Least-squares matrix: full	Hydrogen site location: mixed
*R*[*F*^2^ > 2σ(*F*^2^)] = 0.055	H atoms treated by a mixture of independent and constrained refinement
*wR*(*F*^2^) = 0.150	*w* = 1/[σ^2^(*F*_o_^2^) + (0.1005*P*)^2^] where *P* = (*F*_o_^2^ + 2*F*_c_^2^)/3
*S* = 1.00	(Δ/σ)_max_ < 0.001
4582 reflections	Δρ_max_ = 0.92 e Å^−^^3^
395 parameters	Δρ_min_ = −0.80 e Å^−^^3^
3 restraints	

### Bis[tris(aminothiourea)cobalt(III)] bis[2-(carboxymethyl)-2-hydroxybutanedioato]cobalt(II) tetranitrate tetrahydrate Special details

**Table:**

Geometry. All esds (except the esd in the dihedral angle between two l.s. planes) are estimated using the full covariance matrix. The cell esds are taken into account individually in the estimation of esds in distances, angles and torsion angles; correlations between esds in cell parameters are only used when they are defined by crystal symmetry. An approximate (isotropic) treatment of cell esds is used for estimating esds involving l.s. planes.

### Bis[tris(aminothiourea)cobalt(III)] bis[2-(carboxymethyl)-2-hydroxybutanedioato]cobalt(II) tetranitrate tetrahydrate Fractional atomic coordinates and isotropic or equivalent isotropic displacement parameters (Å^2^)

**Table:**

	*x*	*y*	*z*	*U*_iso_*/*U*_eq_	Occ. (<1)
Co1	0.82571 (3)	0.55594 (6)	0.44027 (6)	0.02392 (19)	
Co2	0.500000	0.500000	0.500000	0.0268 (2)	
S1	0.89780 (4)	0.57221 (10)	0.31121 (9)	0.0299 (3)	
S3	0.78409 (5)	0.72745 (10)	0.35381 (9)	0.0307 (3)	
S2	0.87457 (5)	0.67648 (10)	0.58544 (9)	0.0320 (3)	
O5	0.65342 (13)	0.5523 (3)	0.7133 (3)	0.0348 (7)	
O3	0.53844 (12)	0.3496 (3)	0.5979 (3)	0.0283 (6)	
H3	0.5312 (16)	0.2737 (14)	0.612 (4)	0.042*	
O4	0.58648 (13)	0.5588 (3)	0.5413 (3)	0.0350 (7)	
O6	0.66999 (14)	0.3281 (3)	0.5325 (3)	0.0405 (8)	
O9	0.78502 (15)	0.3036 (3)	0.7225 (3)	0.0419 (8)	
O2	0.50796 (15)	0.6243 (3)	0.8904 (3)	0.0423 (8)	
O14	0.64703 (16)	0.5248 (4)	0.2882 (4)	0.0476 (8)	
H14A	0.633007	0.515855	0.361958	0.071*	
H14B	0.624476	0.577204	0.247038	0.071*	
O1	0.48489 (15)	0.5703 (3)	0.6839 (3)	0.0441 (8)	
N7	0.75933 (15)	0.5446 (3)	0.5529 (3)	0.0285 (7)	
H7A	0.736707	0.480694	0.526268	0.034*	
H7B	0.773626	0.530122	0.635701	0.034*	
O7	0.67078 (16)	0.1366 (3)	0.6050 (4)	0.0467 (8)	
H7	0.659469	0.098157	0.667227	0.070*	
O10	0.78848 (16)	0.3037 (3)	0.9341 (3)	0.0463 (8)	
O15	0.59049 (15)	0.6770 (3)	0.0937 (4)	0.0476 (8)	
H15A	0.567869	0.641229	0.034498	0.071*	
H15B	0.571066	0.730415	0.132922	0.071*	
N1	0.78272 (16)	0.4563 (3)	0.2980 (3)	0.0321 (8)	
H1A	0.783710	0.376846	0.321444	0.038*	
H1B	0.745317	0.480000	0.288731	0.038*	
N8	0.72455 (15)	0.6531 (3)	0.5496 (3)	0.0306 (8)	
H8	0.699347	0.662336	0.606306	0.037*	
N4	0.86476 (15)	0.4111 (3)	0.5347 (3)	0.0304 (8)	
H4A	0.837443	0.363299	0.565348	0.037*	
H4B	0.883111	0.366516	0.477627	0.037*	
N10	0.79107 (16)	0.3600 (3)	0.8299 (3)	0.0332 (8)	
O8	0.79974 (18)	0.4753 (3)	0.8299 (3)	0.0488 (9)	
N2	0.80682 (18)	0.4689 (4)	0.1749 (3)	0.0373 (9)	
H2	0.786582	0.447700	0.103111	0.045*	
O11A	0.9175 (10)	0.224 (2)	0.368 (3)	0.039 (4)	0.5
N11	0.96695 (17)	0.2055 (4)	0.4343 (4)	0.0420 (9)	
N5	0.90574 (18)	0.4465 (4)	0.6407 (4)	0.0391 (9)	
H5	0.926274	0.390669	0.683767	0.047*	
O12A	0.9715 (11)	0.240 (2)	0.542 (2)	0.061 (6)	0.5
N3	0.88378 (19)	0.5169 (4)	0.0582 (4)	0.0404 (9)	
H3A	0.864128	0.490335	−0.012262	0.048*	
H3B	0.918646	0.545588	0.054621	0.048*	
C7	0.60992 (17)	0.5090 (4)	0.6455 (4)	0.0255 (8)	
C6	0.58052 (17)	0.3947 (4)	0.6997 (4)	0.0254 (8)	
O13A	0.9996 (14)	0.114 (3)	0.4205 (19)	0.054 (5)	0.5
N9	0.69505 (18)	0.8363 (4)	0.4571 (4)	0.0452 (10)	
H9A	0.668771	0.842184	0.511769	0.054*	
H9B	0.698370	0.893546	0.399600	0.054*	
N6	0.94963 (19)	0.5926 (5)	0.7769 (4)	0.0523 (12)	
H6A	0.968543	0.534943	0.820948	0.063*	
H6B	0.954741	0.668702	0.799993	0.063*	
C4	0.51204 (19)	0.5526 (4)	0.7954 (4)	0.0313 (9)	
C8	0.62522 (18)	0.2924 (4)	0.7339 (4)	0.0289 (9)	
H8A	0.653819	0.320826	0.803691	0.035*	
H8B	0.605428	0.220686	0.766118	0.035*	
C1	0.86049 (19)	0.5134 (4)	0.1715 (4)	0.0309 (9)	
C9	0.65656 (18)	0.2557 (4)	0.6148 (4)	0.0318 (9)	
C5	0.55003 (18)	0.4372 (4)	0.8193 (4)	0.0284 (9)	
H5A	0.525603	0.370103	0.846255	0.034*	
H5B	0.579699	0.453559	0.891480	0.034*	
C3	0.73011 (18)	0.7399 (4)	0.4613 (4)	0.0295 (9)	
C2	0.91294 (19)	0.5643 (4)	0.6744 (4)	0.0349 (10)	
O12B	0.9840 (10)	0.269 (2)	0.539 (3)	0.052 (5)	0.5
O11B	0.9289 (11)	0.247 (2)	0.353 (3)	0.048 (5)	0.5
O13B	1.0011 (14)	0.134 (3)	0.3756 (19)	0.056 (5)	0.5

### Bis[tris(aminothiourea)cobalt(III)] bis[2-(carboxymethyl)-2-hydroxybutanedioato]cobalt(II) tetranitrate tetrahydrate Atomic displacement parameters (Å^2^)

**Table:**

	*U*^11^	*U*^22^	*U*^33^	*U*^12^	*U*^13^	*U*^23^
Co1	0.0275 (4)	0.0221 (3)	0.0217 (3)	−0.0001 (2)	−0.0001 (2)	0.0010 (2)
Co2	0.0268 (5)	0.0280 (5)	0.0242 (5)	0.0007 (4)	−0.0048 (4)	0.0018 (4)
S1	0.0301 (5)	0.0346 (6)	0.0247 (5)	−0.0025 (4)	0.0014 (4)	−0.0008 (4)
S3	0.0378 (6)	0.0268 (5)	0.0279 (5)	0.0031 (4)	0.0052 (4)	0.0073 (4)
S2	0.0401 (6)	0.0262 (5)	0.0283 (5)	−0.0023 (4)	−0.0036 (4)	−0.0041 (4)
O5	0.0328 (16)	0.0409 (18)	0.0291 (15)	−0.0093 (13)	−0.0050 (12)	−0.0017 (13)
O3	0.0305 (15)	0.0241 (15)	0.0286 (14)	−0.0010 (12)	−0.0057 (11)	0.0024 (12)
O4	0.0317 (16)	0.0369 (18)	0.0345 (16)	−0.0070 (13)	−0.0065 (12)	0.0129 (13)
O6	0.0463 (19)	0.0425 (19)	0.0331 (16)	0.0048 (15)	0.0051 (14)	−0.0001 (15)
O9	0.060 (2)	0.0325 (17)	0.0323 (16)	−0.0073 (15)	0.0012 (14)	−0.0055 (13)
O2	0.056 (2)	0.0397 (19)	0.0302 (16)	0.0132 (16)	−0.0015 (14)	−0.0056 (14)
O14	0.050 (2)	0.050 (2)	0.0435 (19)	−0.0001 (16)	0.0067 (16)	0.0027 (16)
O1	0.052 (2)	0.052 (2)	0.0268 (16)	0.0218 (16)	−0.0045 (14)	−0.0042 (14)
N7	0.0287 (18)	0.0278 (18)	0.0293 (17)	0.0015 (14)	0.0043 (14)	0.0057 (14)
O7	0.057 (2)	0.0302 (18)	0.054 (2)	0.0096 (15)	0.0094 (16)	−0.0059 (15)
O10	0.068 (2)	0.0372 (19)	0.0330 (16)	−0.0033 (16)	0.0030 (15)	0.0072 (14)
O15	0.048 (2)	0.042 (2)	0.051 (2)	0.0017 (16)	−0.0043 (16)	−0.0002 (16)
N1	0.0313 (19)	0.0305 (19)	0.0338 (19)	−0.0047 (15)	−0.0001 (15)	−0.0028 (15)
N8	0.0355 (19)	0.0305 (19)	0.0264 (17)	0.0082 (15)	0.0064 (14)	0.0057 (14)
N4	0.0334 (19)	0.0249 (18)	0.0321 (18)	0.0008 (14)	−0.0019 (14)	−0.0058 (14)
N10	0.036 (2)	0.031 (2)	0.0316 (19)	0.0016 (15)	−0.0021 (15)	0.0011 (16)
O8	0.082 (3)	0.0189 (16)	0.0432 (19)	−0.0046 (16)	−0.0058 (17)	0.0012 (14)
N2	0.048 (2)	0.041 (2)	0.0225 (17)	−0.0086 (17)	−0.0003 (15)	−0.0089 (16)
O11A	0.032 (8)	0.037 (8)	0.047 (8)	−0.006 (6)	0.003 (5)	−0.006 (6)
N11	0.039 (2)	0.036 (2)	0.051 (3)	0.0012 (18)	0.0054 (19)	−0.0076 (19)
N5	0.047 (2)	0.030 (2)	0.037 (2)	0.0117 (17)	−0.0112 (17)	−0.0004 (16)
O12A	0.063 (12)	0.077 (14)	0.038 (7)	0.003 (8)	−0.015 (6)	−0.015 (7)
N3	0.054 (2)	0.040 (2)	0.0283 (19)	−0.0080 (18)	0.0103 (17)	−0.0063 (17)
C7	0.027 (2)	0.027 (2)	0.0228 (19)	0.0032 (16)	0.0032 (16)	−0.0031 (16)
C6	0.0249 (19)	0.025 (2)	0.0254 (19)	−0.0001 (16)	−0.0040 (15)	0.0010 (16)
O13A	0.041 (6)	0.060 (10)	0.061 (12)	0.003 (6)	0.008 (9)	−0.024 (9)
N9	0.055 (3)	0.042 (2)	0.040 (2)	0.021 (2)	0.0136 (18)	0.0125 (18)
N6	0.056 (3)	0.050 (3)	0.047 (2)	0.000 (2)	−0.020 (2)	−0.004 (2)
C4	0.035 (2)	0.032 (2)	0.027 (2)	0.0028 (18)	0.0008 (17)	0.0030 (17)
C8	0.033 (2)	0.028 (2)	0.0257 (19)	0.0080 (17)	−0.0003 (16)	0.0022 (16)
C1	0.038 (2)	0.020 (2)	0.034 (2)	−0.0007 (17)	−0.0008 (18)	0.0002 (16)
C9	0.030 (2)	0.033 (2)	0.031 (2)	0.0044 (17)	−0.0053 (17)	−0.0028 (19)
C5	0.029 (2)	0.027 (2)	0.028 (2)	0.0047 (16)	0.0007 (16)	0.0030 (16)
C3	0.030 (2)	0.032 (2)	0.026 (2)	0.0060 (17)	0.0009 (16)	−0.0007 (17)
C2	0.034 (2)	0.045 (3)	0.025 (2)	0.0021 (19)	0.0003 (17)	−0.0003 (18)
O12B	0.040 (8)	0.048 (8)	0.067 (8)	0.006 (7)	−0.009 (6)	−0.030 (6)
O11B	0.046 (10)	0.056 (10)	0.039 (7)	−0.019 (6)	−0.005 (7)	0.005 (6)
O13B	0.039 (5)	0.064 (12)	0.067 (13)	0.005 (6)	0.021 (10)	−0.029 (10)

### Bis[tris(aminothiourea)cobalt(III)] bis[2-(carboxymethyl)-2-hydroxybutanedioato]cobalt(II) tetranitrate tetrahydrate Geometric parameters (Å, º)

**Table:**

Co1—S1	2.2112 (12)	N1—N2	1.424 (5)
Co1—S3	2.2265 (11)	N8—H8	0.8600
Co1—S2	2.1967 (11)	N8—C3	1.314 (5)
Co1—N7	1.992 (3)	N4—H4A	0.8900
Co1—N1	1.991 (3)	N4—H4B	0.8900
Co1—N4	2.002 (4)	N4—N5	1.416 (5)
Co2—O3	2.060 (3)	N10—O8	1.260 (5)
Co2—O3^i^	2.060 (3)	N2—H2	0.8600
Co2—O4	2.084 (3)	N2—C1	1.322 (6)
Co2—O4^i^	2.084 (3)	O11A—N11	1.28 (3)
Co2—O1	2.081 (3)	N11—O12A	1.15 (2)
Co2—O1^i^	2.081 (3)	N11—O13A	1.25 (3)
S1—C1	1.712 (4)	N11—O12B	1.30 (2)
S3—C3	1.731 (4)	N11—O11B	1.23 (3)
S2—C2	1.707 (5)	N11—O13B	1.29 (3)
O5—C7	1.249 (5)	N5—H5	0.8600
O3—H3	0.852 (9)	N5—C2	1.324 (6)
O3—C6	1.434 (4)	N3—H3A	0.8600
O4—C7	1.264 (5)	N3—H3B	0.8600
O6—C9	1.207 (5)	N3—C1	1.318 (6)
O9—N10	1.249 (5)	C7—C6	1.533 (6)
O2—C4	1.251 (5)	C6—C8	1.524 (5)
O14—H14A	0.8497	C6—C5	1.530 (6)
O14—H14B	0.8500	N9—H9A	0.8600
O1—C4	1.258 (5)	N9—H9B	0.8600
N7—H7A	0.8900	N9—C3	1.313 (6)
N7—H7B	0.8900	N6—H6A	0.8600
N7—N8	1.414 (5)	N6—H6B	0.8600
O7—H7	0.8200	N6—C2	1.314 (6)
O7—C9	1.333 (5)	C4—C5	1.526 (6)
O10—N10	1.231 (5)	C8—H8A	0.9700
O15—H15A	0.8502	C8—H8B	0.9700
O15—H15B	0.8506	C8—C9	1.520 (6)
N1—H1A	0.8900	C5—H5A	0.9700
N1—H1B	0.8900	C5—H5B	0.9700
			
S1—Co1—S3	90.79 (4)	N5—N4—H4B	109.0
S2—Co1—S1	89.58 (4)	O9—N10—O8	119.1 (4)
S2—Co1—S3	86.90 (4)	O10—N10—O9	120.3 (4)
N7—Co1—S1	178.29 (11)	O10—N10—O8	120.6 (4)
N7—Co1—S3	87.60 (10)	N1—N2—H2	120.2
N7—Co1—S2	90.89 (11)	C1—N2—N1	119.7 (3)
N7—Co1—N4	90.30 (15)	C1—N2—H2	120.2
N1—Co1—S1	87.42 (11)	O12A—N11—O11A	116.5 (18)
N1—Co1—S3	89.84 (11)	O12A—N11—O13A	111.1 (18)
N1—Co1—S2	175.53 (11)	O13A—N11—O11A	125.0 (18)
N1—Co1—N7	92.01 (15)	O11B—N11—O12B	120.3 (18)
N1—Co1—N4	95.51 (15)	O11B—N11—O13B	109.0 (18)
N4—Co1—S1	91.36 (11)	O13B—N11—O12B	123.4 (17)
N4—Co1—S3	174.32 (10)	N4—N5—H5	119.5
N4—Co1—S2	87.86 (10)	C2—N5—N4	121.0 (3)
O3^i^—Co2—O3	180.0	C2—N5—H5	119.5
O3^i^—Co2—O4^i^	77.75 (11)	H3A—N3—H3B	120.0
O3—Co2—O4	77.75 (11)	C1—N3—H3A	120.0
O3^i^—Co2—O4	102.25 (11)	C1—N3—H3B	120.0
O3—Co2—O4^i^	102.25 (11)	O5—C7—O4	124.0 (4)
O3^i^—Co2—O1^i^	87.18 (12)	O5—C7—C6	117.1 (3)
O3^i^—Co2—O1	92.82 (12)	O4—C7—C6	118.6 (3)
O3—Co2—O1^i^	92.82 (12)	O3—C6—C7	107.5 (3)
O3—Co2—O1	87.18 (12)	O3—C6—C8	108.1 (3)
O4^i^—Co2—O4	180.0	O3—C6—C5	110.7 (3)
O1^i^—Co2—O4	93.37 (14)	C8—C6—C7	111.0 (3)
O1^i^—Co2—O4^i^	86.63 (14)	C8—C6—C5	112.4 (3)
O1—Co2—O4^i^	93.36 (14)	C5—C6—C7	107.1 (3)
O1—Co2—O4	86.64 (14)	H9A—N9—H9B	120.0
O1^i^—Co2—O1	180.0	C3—N9—H9A	120.0
C1—S1—Co1	97.09 (16)	C3—N9—H9B	120.0
C3—S3—Co1	96.78 (14)	H6A—N6—H6B	120.0
C2—S2—Co1	98.16 (16)	C2—N6—H6A	120.0
Co2—O3—H3	139.7 (18)	C2—N6—H6B	120.0
C6—O3—Co2	108.1 (2)	O2—C4—O1	122.6 (4)
C6—O3—H3	109.2 (16)	O2—C4—C5	117.7 (4)
C7—O4—Co2	111.1 (3)	O1—C4—C5	119.6 (4)
H14A—O14—H14B	104.5	C6—C8—H8A	109.4
C4—O1—Co2	130.4 (3)	C6—C8—H8B	109.4
Co1—N7—H7A	108.9	H8A—C8—H8B	108.0
Co1—N7—H7B	108.9	C9—C8—C6	111.3 (3)
H7A—N7—H7B	107.8	C9—C8—H8A	109.4
N8—N7—Co1	113.1 (2)	C9—C8—H8B	109.4
N8—N7—H7A	108.9	N2—C1—S1	120.3 (3)
N8—N7—H7B	108.9	N3—C1—S1	120.5 (3)
C9—O7—H7	109.5	N3—C1—N2	119.1 (4)
H15A—O15—H15B	109.4	O6—C9—O7	119.5 (4)
Co1—N1—H1A	109.1	O6—C9—C8	123.8 (4)
Co1—N1—H1B	109.1	O7—C9—C8	116.7 (4)
H1A—N1—H1B	107.8	C6—C5—H5A	108.7
N2—N1—Co1	112.6 (3)	C6—C5—H5B	108.7
N2—N1—H1A	109.1	C4—C5—C6	114.4 (3)
N2—N1—H1B	109.1	C4—C5—H5A	108.7
N7—N8—H8	119.4	C4—C5—H5B	108.7
C3—N8—N7	121.1 (3)	H5A—C5—H5B	107.6
C3—N8—H8	119.4	N8—C3—S3	119.9 (3)
Co1—N4—H4A	109.0	N9—C3—S3	120.7 (3)
Co1—N4—H4B	109.0	N9—C3—N8	119.4 (4)
H4A—N4—H4B	107.8	N5—C2—S2	119.9 (3)
N5—N4—Co1	113.0 (3)	N6—C2—S2	121.1 (4)
N5—N4—H4A	109.0	N6—C2—N5	119.0 (4)
			
Co1—S1—C1—N2	−4.9 (4)	O3—C6—C8—C9	59.8 (4)
Co1—S1—C1—N3	172.1 (3)	O3—C6—C5—C4	−68.1 (4)
Co1—S3—C3—N8	−4.3 (4)	O4—C7—C6—O3	14.4 (5)
Co1—S3—C3—N9	174.6 (4)	O4—C7—C6—C8	132.4 (4)
Co1—S2—C2—N5	0.1 (4)	O4—C7—C6—C5	−104.6 (4)
Co1—S2—C2—N6	−179.7 (4)	O2—C4—C5—C6	−147.9 (4)
Co1—N7—N8—C3	12.1 (5)	O1—C4—C5—C6	34.2 (6)
Co1—N1—N2—C1	17.5 (5)	N7—N8—C3—S3	−4.4 (5)
Co1—N4—N5—C2	3.9 (6)	N7—N8—C3—N9	176.6 (4)
Co2—O3—C6—C7	−37.4 (3)	N1—N2—C1—S1	−7.5 (6)
Co2—O3—C6—C8	−157.3 (3)	N1—N2—C1—N3	175.4 (4)
Co2—O3—C6—C5	79.3 (3)	N4—N5—C2—S2	−2.6 (6)
Co2—O4—C7—O5	−158.1 (3)	N4—N5—C2—N6	177.1 (4)
Co2—O4—C7—C6	16.3 (4)	C7—C6—C8—C9	−57.8 (4)
Co2—O1—C4—O2	160.6 (4)	C7—C6—C5—C4	48.8 (4)
Co2—O1—C4—C5	−21.6 (7)	C6—C8—C9—O6	36.7 (6)
O5—C7—C6—O3	−170.8 (3)	C6—C8—C9—O7	−145.1 (4)
O5—C7—C6—C8	−52.8 (5)	C8—C6—C5—C4	170.9 (4)
O5—C7—C6—C5	70.2 (4)	C5—C6—C8—C9	−177.7 (3)

Symmetry code: (i) −*x*+1, −*y*+1, −*z*+1.

### Bis[tris(aminothiourea)cobalt(III)] bis[2-(carboxymethyl)-2-hydroxybutanedioato]cobalt(II) tetranitrate tetrahydrate Hydrogen-bond geometry (Å, º)

**Table:**

*D*—H···*A*	*D*—H	H···*A*	*D*···*A*	*D*—H···*A*
O14—H14*B*···O15	0.85	2.00	2.798 (5)	157
N7—H7*A*···O6	0.89	2.25	3.100 (5)	159
N7—H7*B*···O8	0.89	2.10	2.983 (5)	172
O7—H7···O14^ii^	0.82	1.85	2.648 (5)	163
O15—H15*A*···O2^iii^	0.85	1.92	2.730 (5)	158
O15—H15*B*···O4^iv^	0.85	2.50	2.902 (5)	110
N1—H1*A*···O9^v^	0.89	2.20	2.912 (5)	137
N1—H1*A*···O10^v^	0.89	2.26	3.128 (5)	165
N1—H1*B*···O14	0.89	2.30	3.185 (5)	172
N8—H8···O5	0.86	1.98	2.670 (5)	136
N4—H4*A*···O9	0.89	2.19	3.000 (5)	152
N4—H4*A*···O10^v^	0.89	2.45	3.024 (5)	122
N4—H4*B*···O11*A*	0.89	2.09	2.96 (3)	166
N4—H4*B*···O12*A*	0.89	2.48	3.06 (3)	123
N4—H4*B*···O12*B*	0.89	2.56	3.13 (2)	122
N4—H4*B*···O11*B*	0.89	2.15	3.04 (3)	175
N2—H2···O10^iii^	0.86	2.32	3.032 (5)	140
N5—H5···O11*A*^ii^	0.86	2.28	2.96 (3)	136
N5—H5···O12*A*	0.86	2.47	2.92 (3)	114
N5—H5···O12*B*	0.86	2.45	2.88 (2)	112
N5—H5···O11*B*^ii^	0.86	2.27	3.02 (3)	145
N3—H3*A*···O8^iii^	0.86	2.08	2.913 (5)	161
N3—H3*B*···O13*A*^vi^	0.86	2.01	2.86 (3)	170
N3—H3*B*···O13*B*^vi^	0.86	2.13	2.94 (3)	157
N9—H9*A*···O15^vii^	0.86	2.06	2.883 (6)	161
N9—H9*B*···O5^iv^	0.86	2.15	2.844 (5)	137
N6—H6*A*···O13*A*^ii^	0.86	2.00	2.85 (3)	170
N6—H6*A*···O13*B*^ii^	0.86	2.03	2.86 (3)	161
N6—H6*B*···O12*A*^viii^	0.86	2.35	2.93 (3)	125
N6—H6*B*···O12*B*^viii^	0.86	2.32	2.99 (3)	134

Symmetry codes: (ii) *x*, −*y*+1/2, *z*+1/2; (iii) *x*, *y*, *z*−1; (iv) *x*, −*y*+3/2, *z*−1/2; (v) *x*, −*y*+1/2, *z*−1/2; (vi) −*x*+2, *y*+1/2, −*z*+1/2; (vii) *x*, −*y*+3/2, *z*+1/2; (viii) −*x*+2, *y*+1/2, −*z*+3/2.
